# Protective Effect of Caffeic Acid Derivatives on *tert*-Butyl Hydroperoxide-Induced Oxidative Hepato-Toxicity and Mitochondrial Dysfunction in HepG2 Cells

**DOI:** 10.3390/molecules22050702

**Published:** 2017-04-28

**Authors:** Tzung-Hsun Tsai, Chun-Hsien Yu, Yu-Ping Chang, Yu-Ting Lin, Ching-Jang Huang, Yueh-Hsiung Kuo, Po-Jung Tsai

**Affiliations:** 1 Department of Dentistry, Keelung Chang-Gung Memorial Hospital, Keelung 204, Taiwan; tts1725@gmail.com; 2 Department of Pediatrics, Taipei Tzu-Chi Hospital, Buddhist Tzu-Chi Medical Foundation, New Taipei City 231, Taiwan; chryu@mail.tcu.edu.tw; 3 Department of Pediatrics, College of Medicine, Buddhist Tzu-Chi University, Hualien 970, Taiwan; 4 Department of Human Development and Family Studies, National Taiwan Normal University, Taipei 106, Taiwan; elf_elisa@yahoo.com.tw (Y.-P.C.); linyuting830812@gmail.com (Y.-T.L.); 5 Institute of Microbiology and Biochemistry, and Department of Biochemical Science and Technology, National Taiwan University, Taipei 106, Taiwan; cjjhuang@ntu.edu.tw; 6 Department of Chinese Pharmaceutical Sciences and Chinese Medicine Resources, China Medical University, Taichung 404, Taiwan; 7 Department of Biotechnology, Asia University, Taichung 413, Taiwan

**Keywords:** caffeic acid derivatives, *tert*-butyl hydroperoxide, antioxidant, liver protection

## Abstract

Oxidative stress results in structural and functional abnormalities in the liver and is thought to be a crucial factor in liver diseases. The aim of this study was to investigate the cytoprotective and antioxidant effects of caffeic acid (CA) derivatives on *tert*-butyl hydroperoxide (*t*-BHP)-induced oxidative stress in HepG2 cells. Nine CA derivatives were synthesized, including *N*-phenylethyl caffeamide (PECA), *N*-(3-florophen)methyl caffeamide (FMCA), *N*-(4-methoxy-phen)methyl caffeamide (MPMCA), *N*-heptyl caffeamide (HCA), *N*-octyl caffeamide (OCA), octyl caffeate (CAOE), phenpropyl caffeate (CAPPE), phenethyl caffeate (CAPE), and phenmethyl caffeate (CAPME). The results showed that CA and its derivatives significantly inhibited *t*-BHP-induced cell death of HepG2 cells. The rank order of potency of the CA derivatives for cytoprotection was CAOE > HCA > OCA > FMCA > CAPPE > CAPME > CAPE > PECA > MPMCA > CA. Their cytoprotective activity was associated with lipophilicity. The antioxidant effect of these compounds was supported by the reduction in the levels of thiobarbituric acid reactive substrates, a biomarker of lipid peroxidation, in HepG2 cells. Pre-treatment of CA derivatives significantly prevented the depletion of glutathione, the most important water-soluble antioxidant in hepatocytes. Pre-treatment of CA derivatives before *t*-BHP exposure maintained mitochondrial oxygen consumption rate and ATP content in the injured HepG2 cells. CA derivatives except OCA and HCA significantly suppressed *t*-BHP-induced hypoxia-inducible factor-1α (HIF-1α) protein level. In addition, all of these CA derivatives markedly increased the nuclear factor erythroid 2-related factor 2 (Nrf2) accumulation in the nucleus, indicating that their cytoprotection may be mediated by the activation of Nrf2. Our results suggest that CA derivatives might be a hepatoprotective agent against oxidative stress.

## 1. Introduction

Increased oxidative stress and associated high levels of free radical generation have been described to occur during the pathogeneses of various diseases. Excessive reactive oxygen species (ROS) production plays a crucial role in liver diseases, including paracetamol-induced liver damage, alcoholic liver disease, nonalcoholic steatohepatitis, liver cirrhosis and fibrosis [1,2]. Chronic liver inflammation leads to oncogenic mutations in numerous cellular signaling cascades. Elevated levels of ROS, induced by hepatitis viruses, are considered as a key factor underlying the oncogenic effects of hepatitis B and C viruses [3,4]. Oxidative damage is the most likely causative process to alter and deplete mitochondria DNA, stimulate apoptotic pathways, and increase the tendency for hepatocellular necrosis [5]. Therefore, antioxidant therapy alone or in combination with other pharmacological strategies has been proposed as a reasonable treatment of a variety of liver diseases.

**Figure 1:**
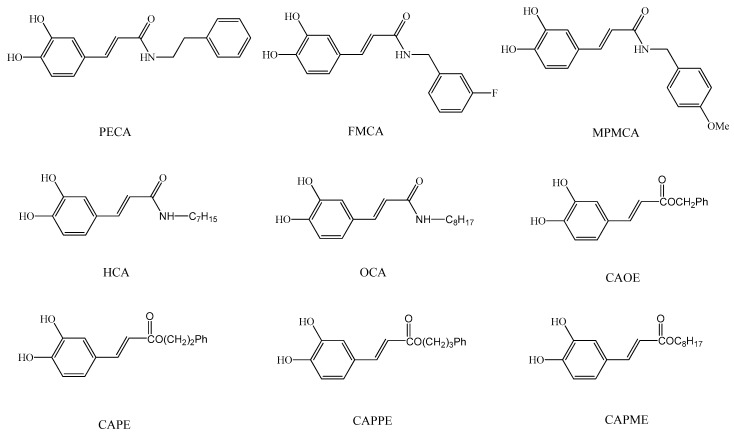
Chemical structures of caffeic acid derivatives.

Caffeic acid (CA) occurs widely in food plants and is thus widely consumed in the human diet. CA is absorbed in humans after oral intake and specific metabolites can be detected in the urine [6]. CA exerts various biological activities, such as anti-oxidation, anti-inflammatory, and immune regulation effects [7]. In addition, its derivatives also show unique pharmaceutical functions. For example, the phenethyl ester of caffeic acid (CAPE), an active component of honeybee propolis extract, possesses antimicrobial, antioxidant, anti-inflammatory, antitumoral, and neuroprotective properties [8]. Due to their potential health benefits, CA and its derivatives have attracted considerable interest in the past few years. As a result, CA has been widely used as a template for the development of new chemical entities with a potential therapeutic interest in human diseases associated with oxidative stress [9]. As the synthesis of esters, amides and hybrids base on the CA scaffold could yield new or effective agents against oxidative stress, we prepared nine CA derivatives using CA as a substrate, including *N*-phenylethyl caffeamide (PECA), *N*-(3-florophen)methyl caffeamide (FMCA), *N*-(4-methoxyphen)methyl caffeamide (MPMCA), *N*-heptyl caffeamide (HCA), *N*-octyl caffeamide (OCA), octyl caffeate (CAOE), phenpropyl caffeate (CAPPE), phenethyl caffeate (CAPE; phenethyl ester of caffeic acid), and phenmethyl caffeate (CAPME) in this study (Figure 1).

*tert*-Butyl hydroperoxide (*t*-BHP), a pro-oxidant, induces oxidative stress and cell injury that result from the intracellular production of ROS. *t*-BHP has been used as a model compound to investigate the mechanisms of cell damage initiated by oxidative stress. In hepatocyte cultures and livers, *t*-BHP can be metabolized to free radical intermediates, and these intermediates result in initiating lipid peroxidation, decreasing mitochondrial membrane potential, changes in mitochondrial membrane integrity, depletion of cellular stores of glutathione (GSH), damage to cell integrity and hepatic inflammation [10,11,12].

HepG2 cells have been considered to be a good tool to study the in vitro xenobiotic metabolisms, and cytoprotective, genotoxic, and antigenotoxic effects of compounds, as they retain the activity of many phase I, phase II, and antioxidant enzymes [13,14,15,16]. Here, we evaluated the protective effects of the nine CA derivatives on *t*-BHP-induced oxidative injury and mitochondrial dysfunction in HepG2 cells.

## 2. Results

### 2.1. Protective Effect of CA Derivatives against t-BHP Induced Cytotoxicity

**Figure 2:**
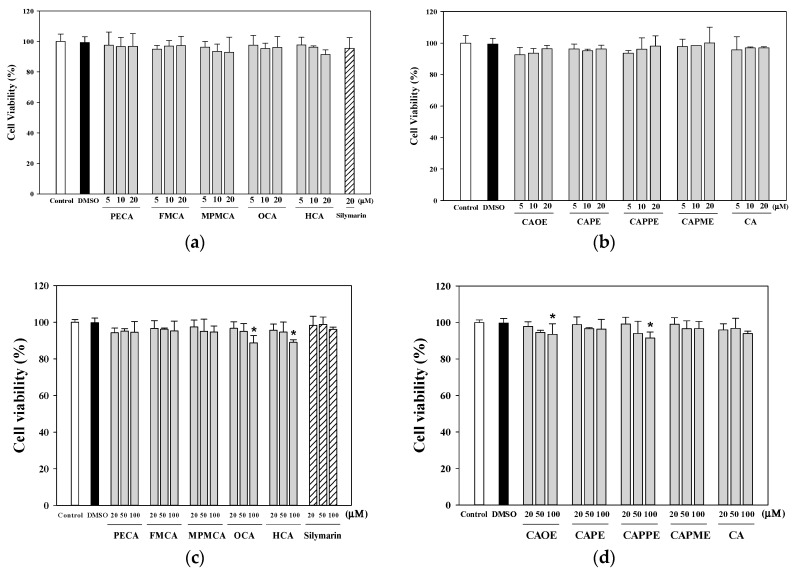
Effect of caffeic acid derivatives on HepG2 cell viability. HepG2 cells were treated with different concentration of samples for 24 h (**a**,**b**) and 72 h (**c**,**d**). Cell viability is expressed as the percentage of vehicle control (0.1% DMSO). Control, medium alone without DMSO. Values were shown as mean ± SD (*n* = 3). *, *p* < 0.05 vs. DMSO-vehicle.

Treatment of the cells with CA and its derivatives (5, 10 and 20 μM) for 24 h (Figure 2a,b) had no apparent cytotoxicity. When the HepG2 cells treated for 72 h with increasing concentrations of CA and its derivatives, OCA, HCA, CAOE, and CAPPE (100 μM) significantly decreased the cell viability (Figure 2c,d). Then, we chose non-toxic concentrations (1–20 μM) for all subsequent experiments. The cytoprotection effect of CA derivatives against *t*-BHP induced cell death was evaluated by pre-incubating the cells with CA derivatives for 24 h, followed by treatment with 0.7 mM of *t*-BHP for 24 h. Silymarin (20 μM) was used as a control.

**Figure 3:**
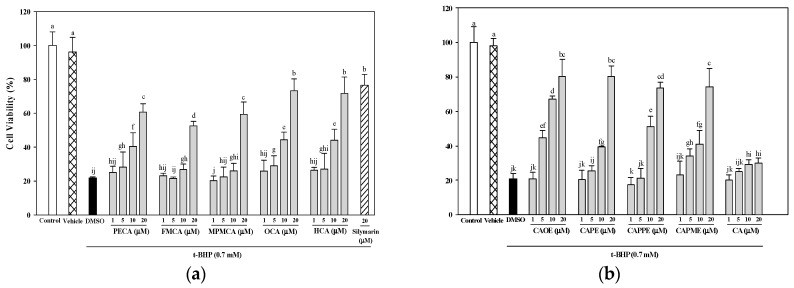
Protective effects of caffeic acid derivatives of *t*-BHP-induced cytotoxicity. HepG2 cells were treated with different concentrations of caffeamide derivatives (**a**) and caffeate derivatives (**b**) for 24 h before being exposed to *t*-BHP (0.7 mM) for 20 h. The vehicle and positive controls respectively received DMSO (0.1%) and silymarin (20 μM). Cell viability is expressed as the percentage of control. Values were shown as mean ± SD (*n* = 3). Data were analyzed by one-way ANOVA and Duncan multiple comparison test. Values not sharing the same letter are significantly different at *p* < 0.05.

As shown in Figure 3, all CA derivatives (10 and 20 μM) showed significant cytoprotection effects against *t*-BHP-induced cell death. At the concentration of 20 μM, FECA was the least effective in inhibiting *t*-BHP-mediated toxicity and was more potent than silymarin. CA was less efficient and did not achieve the IC_50_ in the experiments with *t*-BHP up to the highest concentration tested (20 μM). All CA derivatives were more potent than CA (Figure 3). The potency of the CA and its derivatives was expressed as IC_50_ values. The rank order of potency of them for cytoprotection was CAOE (5.9 μM) > HCA (11.4 μM) > OCA (11.7 μM) > FMCA (11.9 μM) > CAPPE (12.1 μM) > CAPME (12.4 μM) > CAPE (12.7 μM) > PECA (14.1 μM) > MPMCA (18.8 μM) > CA (not applicable).

### 2.2. Effects of CA Derivatives on t-BHP-Induced Lipid Peroxidation

The thiobarbituric acid reactive substrates (TBARS) assay is still the most commonly used assay for the measurement of lipid peroxidation in biomaterials. It is relatively simple, fast, and cost-effective.

**Figure 4:**
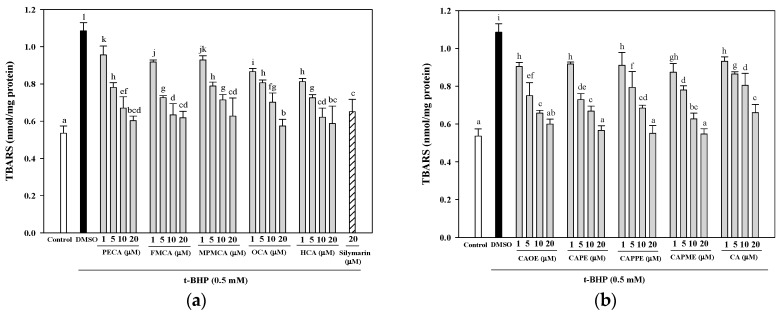
Inhibitory effects of caffeic acid derivatives on *t*-BHP-induced lipid peroxidation in HepG2 cells. Pre-treatment with caffeamide derivatives (**a**) and caffeate derivatives (**b**) for 24 h, HepG2 cells were stimulated with *t*-BHP (0.5 mM) for 24 h. The vehicle and positive controls respectively received DMSO (0.1%) and silymarin (20 μM). The amount of TBARS formation is expressed as nmol/mg protein. Values were shown as mean ± SD (*n* = 3). Data were analyzed by one-way ANOVA and Duncan multiple comparison test. Values not sharing the same letter are significantly different at *p* < 0.05.

As shown in Figure 4, the effect of CA derivatives on TBARS formation in t-BHP-treated HepG2 cells was examined. Treatment of HepG2 cells with 0.5 mM t-BHP for 24 h resulted in a significant increase in TBARS concentration. However, pretreatment with the CA derivatives (at concentrations of 5, 10, and 20 μM) effectively inhibited the increase in TBARS production induced by t-BHP. Pre-treatments with 20 μM of PECA, FMCA, MPMCA, OCA, HCA, CAOE, CAPE, CAPPE, and CAPME reduced t-BHP-induced TBARS formation by 44.4%, 43.0%, 42.2%, 47.1%, 45.8%, 44.7%, 47.9%, 49.2%, and 49.6%, respectively. Five CA derivatives (20 μM), including OCA, CAOE, CAPE, CAPPE, and CAPME, are more potent than silymarin (20 μM) in the inhibition of t-BHP-induced lipid peroxidation.

### 2.3. Effects of CA Derivatives on Glutathione Levels

**Figure 5:**
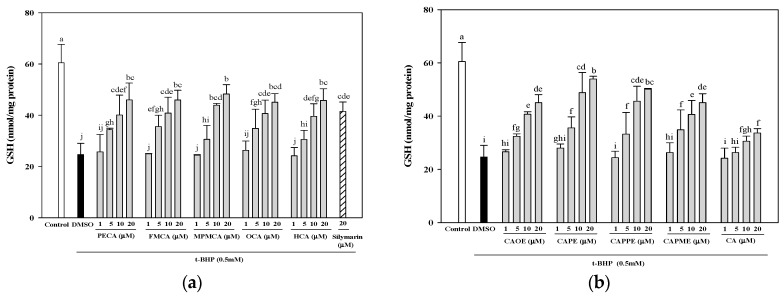
Effects of caffeic acid derivatives on the glutathione levels in HepG2 cells after the oxidative damage induced by *t*-BHP. Pre-treatment with caffeamide derivatives (**a**) and caffeate derivatives (**b**) for 24 h, HepG2 cells were stimulated with *t*-BHP (0.5 mM) for 24 h. The vehicle and positive controls respectively received DMSO (0.1%) and silymarin (20 μM). The amount of cellular glutathione level is expressed as nmol/mg protein. Values were shown as mean ± SD (*n* = 3). Data were analyzed by one-way ANOVA and Duncan multiple comparison test. Values not sharing the same letter are significantly different at *p* < 0.05.

Treatment with 0.5 mM *t*-BHP induced a remarkable decrease in GSH levels compared to the control and DMSO vehicle (60.5 and 24.7 nmol/mg protein, respectively). Pre-treatments with 20 μM of PECA, FMCA, MPMCA, OCA, HCA, CAOE, CAPE, CAPPE, and CAPME prevented GSH depletion by 76.0%, 76.0%, 79.8%, 74.5%, 75.7%, 74.5%, 89.2%, 82.8%, and 74.5%, respectively (Figure 5). Among the tested compounds, CAPE presented the highest efficiency in a concentration-dependent manner (Figure 5).

### 2.4. Effects of CA Derivatives on t-BHP-Induced Mitochondrial Dysfunction

**Figure 6:**
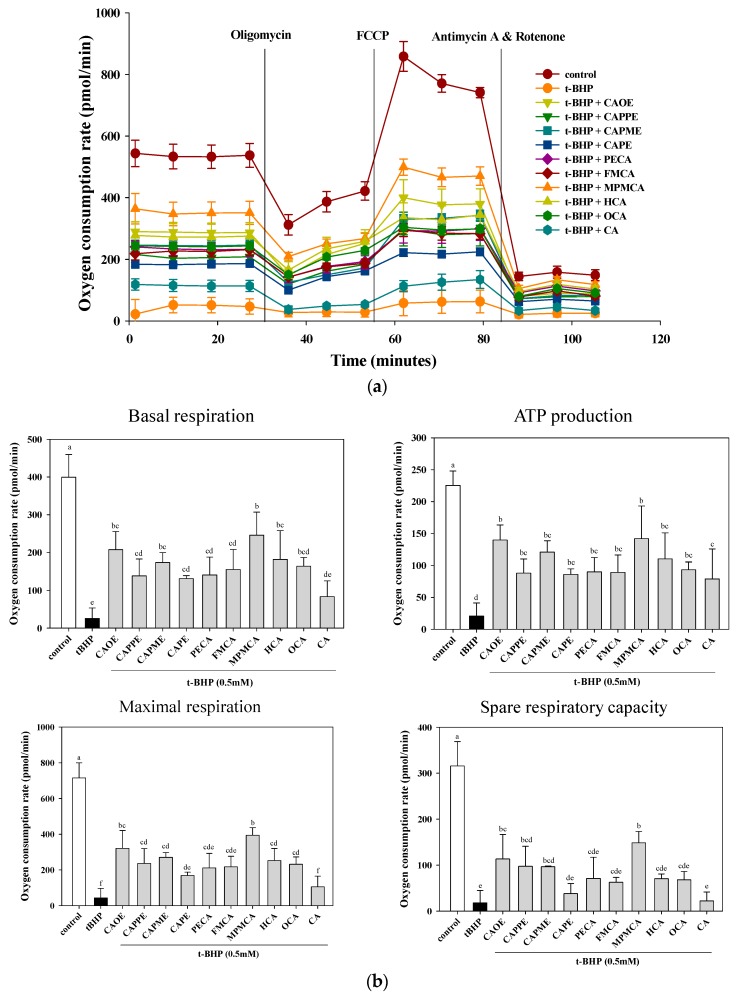
Effect of CA derivatives on *t*-BHP-induced mitochondria dysfunction. HepG2 cells were treated with CA and CA derivatives (20 μM) or control (vehicle, 0.1% DMSO) for 6 h, followed by 0.5 mM *t*-BHP for an additional 2 h. Respiration curve (**a**) and statistical analyses of basal, maximal and spare mitochondrial respiration capacity, and ATP production (**b**) were analyzed. Values were shown as mean ± SD (*n* = 3). Data were analyzed by one-way ANOVA and Duncan multiple comparison test. Values not sharing the same letter are significantly different at *p* < 0.05.

Further investigation on mitochondrial oxygen consumption was conducted with CA derivatives at the concentration of 20 μM. As shown in Figure 6, *t*-BHP abolished mitochondrial respiration capacity including basal, maximal, and spare respirations as well as ATP production, all of which were significantly improved by CA derivatives pre-treatment. In contrast, at the same concentrations, CA is ineffective in maintaining basal, maximal, and spare respirations. The CA derivatives are superior to CA in protecting against oxidant-induced mitochondrial dysfunction.

### 2.5. Effects of CA Derivatives on HIF-1α and Nrf2 Levels

**Figure 7:**
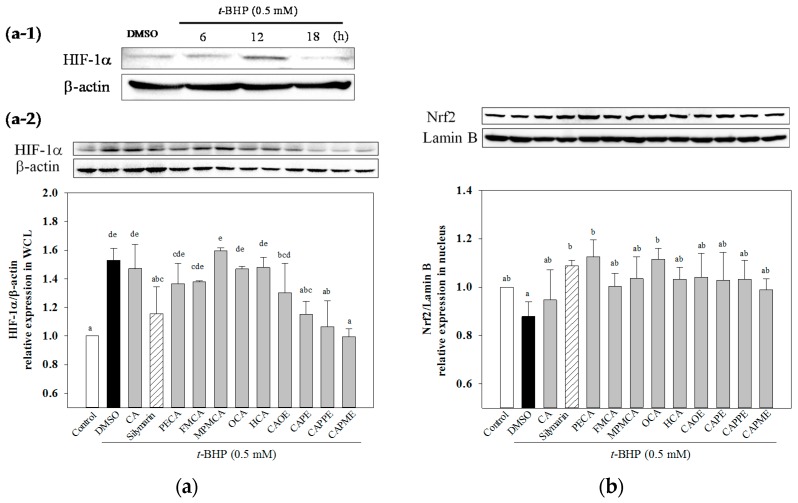
Effect of CA derivatives on HIF-1α and Nrf2 protein levels in *t*-BHP-treated HepG2 cells. Cells were treated with *t*-BHP (0.5 mM) alone for the indicated times (**a**-**1**). Pre-treatment with test compounds (20 μM) for 24 h, cells were then stimulated with *t*-BHP (0.5 mM) for 12 h (**a**-**2**). The equal amounts of whole cell lysate (WCL) were subjected to immunoblotting to determine the HIF-1α level. Quantification of protein levels was normalized to β-actin (**a**). Pre-treatment with test compounds (20 μM) for 24 h, cells were treated with *t*-BHP (0.5 mM) for 12 h. Then, equal amounts of nuclear protein were subjected to western blot analysis to determine the Nrf2 level. Quantification of protein levels was normalized to lamin B (**b**). The vehicle and positive controls respectively received DMSO (0.1%) and silymarin (20 μM). Results were expressed as mean ± SD of three experiments. Data were analyzed by one-way ANOVA and Duncan multiple comparison test. Values not sharing the same letter are significantly different at *p* < 0.05.

Next, we determined whether *t*-BHP affects hypoxia-inducible factor-1α (HIF-1α) protein level. As shown in Figure 7a-1, HIF-1α protein expression was markedly induced by *t*-BHP at 12 h and then declined. All of these CA derivatives except OCA and HCA significantly inhibited the *t*-BHP-induced HIF-1α protein levels. While CA did not significantly attenuate HIF-1α expression induced by *t*-BHP (Figure 7a-2). The translocation of nuclear factor erythroid-2-related factor (Nrf2) from the cytosol to nucleus is a key step for Nrf2 activation [17]. Thus, we explore whether CA derivatives could increase Nrf2 accumulation in the nucleus. As shown in Figure 7b, when HepG2 cells were treated with *t*-BHP for 12 h, *t*-BHP significantly decreased nuclear Nrf2 level as compared to the untreated control cells. Pretreatment with CA and silymarin (20 μM) markedly increased Nrf2 accumulation in the nucleus. All of these CA derivatives (20 μM) also significantly enhanced nuclear Nrf2 level when cells were incubated with *t*-BHP for 12 h (Figure 7b). Our data demonstrated that nuclear Nrf2 expression was enhanced by CA derivatives in *t*-BHP-treated HepG2 cells.

## 3. Discussion

Numerous studies have demonstrated the biological and pharmacological actions of CA derivatives, especially CAPE. CAPE is a potent antioxidant, which effectively scavenges ROS and protects the cell membrane against lipid peroxidation [8]. Moreover, CAPE possesses hepato-protective activity against tetrachloride or cholestasis-induced liver injury [18,19] and *t*-BHP-induced cytotoxicity in HepG2 cells and rat liver [20]. In addition, CAOE exerts anti-virus activity against hepatitis C virus, which is a major causative agent of chronic liver disease including cirrhosis and hepatocellular carcinoma [21]. Caffeamide derivatives are more stable compound than CAPE and could potentially extend the beneficial effects of CAPE [22]. In particular, PECA prevents photo-damage and protects against the metabolic consequences of diabetes mellitus [23,24]. Several catechol ring-fluorinated derivatives of CA phenethylamide show cytoprotective activity against H_2_O_2_ induced oxidative stress in human umbilical vein endothelial cells [25]. To the best of our knowledge, there is no study relating the biological and pharmacological activities of FMCA, MPMCA, HCA, OCA, and CAPME. Moreover, the information concerning the hepato-protective action of CA derivatives is still limited, with the exception of CAPE. Therefore, the possible cytoprotective effect of the nine CA derivatives against *t*-BHP-induced cell death of hepatocytes was investigated in this study.

Silymarin displays antioxidant, anti-inflammatory, and immunomodulatory actions [26] and has been widely used as a therapeutic agent for a variety of acute and chronic liver diseases. Silymarin reverses the GSH content and reduces lipid peroxides (malondialdehyde, MDA) in isolated rat hepatocytes and human erythrocytes that were altered by *t*-BHP [27,28,29]. Due to its proven hepatoprotective and antioxidant properties, we used silymarin as a positive control in this study. Death of liver cells is a characteristic feature of many liver diseases, such as cholestasis, hepatitis, or ischemia/reperfusion [30]. In this study, all nine CA derivatives exerted similar effective cytoprotection as compared to silymarin (Figure 3). In addition, cells treated with the nine CA derivatives showed higher protective effect compared to cells treated with CA against oxidative cell injury (Figure 3). Interestingly, CA-derived ester analogues are decomposed by esterases that lead to their low bioavailability in vivo [31]. However, CAOE exhibited the most potent cytoprotective activity in this study. Concerning structure activity relationship, Wu et al. [32] reported that the antioxidative activity of CAPE and its related compounds (CA, ferulic acid, and ethyl ferulate) depends on the hydrogen groups or catechol rings, the polarity, hydrophobicity (or partitioning properties between lipid and aqueous phases) and stability of the antioxidants. All CA derivatives used in this work have the same *ortho*-dihydroxyl functionality in the catechol ring. In our present experimental condition, the crucial structural feature responsible for the better cytoprotection activities of CAOE, OCA and HCA is their hydrophobic side chains. For CAOE, OCA and HCA, the conversion of the acid group to the ester or amide group decrease the molecular polarity. The higher lipophilicity might facilitate the transport of these compounds through cell membrane and reach cell interior, which then these compounds could scavenge the ROS where they were produced.

Next, investigation of whether cytoprotective action of CA derivatives is related to the antioxidant capacity was conducted. Oxidative stress is an imbalance between pro-oxidants and anti-oxidants. Overproduction of ROS or inadequate antioxidant defenses (e.g., low levels of vitamins, GSH), or both, can lead to liver injury. Intracellular MDA concentration is a direct result of membrane unsaturated fatty acid peroxidation and was used as a biomarker for *t*-BHP-induced oxidation in HepG2 cells. All CA derivatives efficiently inhibited the TBARS formation (Figure 4). Moreover, GSH depletion is considered a potential biomarker of drug-induced hepatotoxicity. Because hepatic mitochondria lack catalase, GSH plays a critical role in protecting mitochondria against oxidative stress [33]. Our data demonstrated that all CA derivatives prevented GSH depletion in *t*-BHP-treated HepG2 cells (Figure 5).

Hepatocytes are normally rich in mitochondria and each hepatocyte contains about 800 mitochondria occupying about 18% of the entire liver cell volume. Mitochondria play an important role in hepatocyte metabolism and ATP production. The mitochondrial dysfunction contributes to oxidative stress. Oxidative stress is believed to play an important role in the development of steatosis and liver lesion [34]. We further examined whether CA derivatives preserve mitochondrial respiration and energy production capacity in the presence of *t*-BHP. Mitochondrial metabolic function was determined by measuring mitochondrial oxygen consumption rate (OCR) using an XF24 analyzer. After *t*-BHP treatment, OCR of HepG2 cells rapidly declined, confirming mitochondrial dysfunction (Figure 6) and consistent with previous studies in myotubes [35]. Pre-treatment with CA derivatives preserved mitochondrial OCR in *t*-BHP-treated cells (Figure 6). All CA derivatives herein prevent mitochondrial dysfunction and energy deficits caused by oxidant exposure. Mitochondrial dysfunction is a common mechanism in the etiology of organ injuries and diseases caused by metabolic insufficiency. Our results highlight the potential of CA derivatives to treat disorders characterized by mitochondrial impairment.

HIF-1α is a heterodimeric transcription factor that plays a key role in the signaling pathways transmitting information regarding cellular oxygen levels. There is evidence to suggest that ROS regulate HIF-1α stabilization under normoxia. Addition of exogenous H_2_O_2_ stabilizes HIF-1α protein under normoxia [36]. After 12 h treatment of *t*-BHP, an exogenous inducer of oxidative stress, increased HIF-1α protein level (Figure 7a-1). While, CA derivatives except OCA and HCA significantly decreased *t*-BHP-induced HIF-1α protein level under normoxia (Figure 7a-2). However, the mechanisms explaining how HIF-1α could mediate *t*-BHP-induced damage remains unclear. Nrf2 signal plays a crucial role against oxidative stress and cytotoxicity. The enhanced activation of Nrf2 exerts hepatoprotective activity in different oxidative stress models, such as acetaminophen or CCl_4_-induced liver injury [5,37]. Activation of Nrf2 protects mitochondria from oxidative stress [5]. CAPE has been shown to protect liver cells against CCl_4_-induced oxidative stress and inhibit activation of hepatic stellate cells through up-regulated Nfr2 expression [38]. In this study, we demonstrated that all CA derivatives increased nuclear Nrf2 accumulation (Figure 7b). Taken together, these findings suggested that Nrf2 signaling pathways could play a key role in mediating the hepatoprotective effects of CA derivatives.

In conclusion, our data suggested that CA derivatives have protective effects against oxidant-induced hepatotoxicity, at least in part, by attenuation of lipoperoxidation, preservation of glutathione, inhibition of mitochondrial dysfunction, and regulation of the master regulator of antioxidant response Nrf2. However, further studies are still required to validate the detailed molecular mechanisms involved in the protective effect of CA derivatives.

## 4. Materials and Methods

### 4.1. General Information

All chemicals, including caffeic acid, *N,N*-dimethylformamide, trimethylamine, BOP, CH_2_Cl_2_, EtOAc, HCl, thionyl chloride, SOCl_2_, NaHCO_3_ (aq.), and MgSO_4_, were of analytical-grade purity. They were purchased from the branch of Merck in Taipei, Taiwan, and the branch of Sigma Aldrich in Taichung, Taiwan. UV spectra were recorded on a Shimadzu UV-1601PC spectrophotometer. Infrared (IR) spectra were measured on a Perkin-Elmer-983G FT-IR spectrophotometer. ^1^H- and ^13^C-NMR and 2D NMR spectra were obtained on a Bruker DRX-500 FT-NMR spectrometer with tetramethylsilane (TMS) as the internal standard. EI-MS were measured with a Jeol-JMSHX300 mass spectrometer. Silica gel (230–400 mesh; Merck & Co., Inc.) was used for column chromatography (CC). Pre-coated silica gel (60 F-254; Merck & Co., Inc.) plates were used for TLC. The spots on TLC were detected by spraying with 5% H_2_SO_4_ and then heating at 100 °C.

### 4.2. Synthesis of Caffeamide Derivatives

**Scheme 1:**
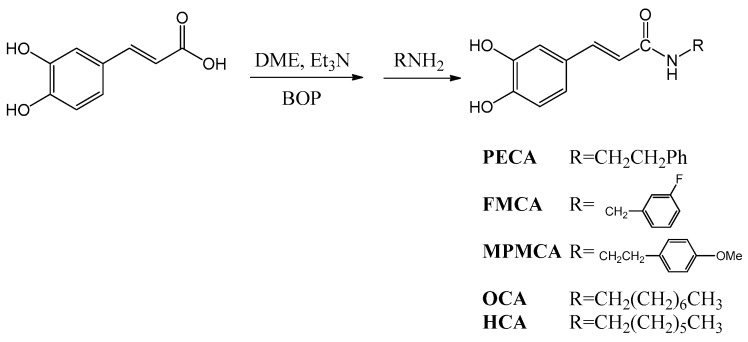
Synthesis of caffeic acid amide derivatives.

Compounds were obtained by the following method: caffeic acid (100 mg, 0.56 mmol) was dissolved in the mixture of *N*,*N*-dimethylformamide (DMF, 1 mL) and trimethylamine (80 μL), and then the appropriate amine derivative (80 μL, assumes that they all have the same density and molecular weight, 1.2 eq.) was added to the reaction solution cooled in an ice bath (0 °C), and then BOP (295 mg, 1.2 eq.) in CH_2_Cl_2_ (5 mL) was added to the reaction mixture, followed by stirring for 30 min (Scheme 1). The reaction mixture was kept at room temperature for 2 h. The solvent was then evaporated under vacuum, and the crude mixture was partitioned between EtOAc and H_2_O. The organic layer was washed with 3 N aqueous HCl and then with 10% aqueous NaHCO_3_ solution. The product was purified by chromatography on silica gel. The yields of **PECA**, **FMCA**, **MPMCA**, **OCA**, and **HCA** were 70%~80%.

*N-Phenylethyl caffeamide* (**PECA**): Pale white needle-like crystals; m.p.: 148–149 °C; IR ν_max_ (cm^−1^): 3288, 1642, 1591, 1523, 1361, 1279, 1036, 975, 849. ^1^H-NMR (CD_3_COCD_3_, 500 MHz): δ 2.84 (2H, t, *J* = 6.8 Hz), 3.53 (2H, q, *J* = 6.8 Hz), 6.43 (1H, d, *J* = 15.2 Hz), 6.83 (1H, d, *J* = 8.1 Hz), 6.92 (1H, dd, *J* = 8.1, 1.8 Hz), 7.07 (1H, d, *J* = 1.8 Hz), 7.15–7.30 (5H, m), 7.35 (-NH, br. s), 7.43 (1H, d, *J* = 15.2 Hz), 8.20 (-OH, s), 8.42 (-OH, s). EI-MS *m*/*z* (%): 283 (M^+^, 17), 178 (22), 163 (100); UV (MeOH) λ_max_ (logε): 322 (4.42), 296 (4.36), 245 (4.30), 216 (4.61)nm.

*N-(3-Florophen)methyl caffeamide* (**FMCA**): White solid; m.p.: 186–188 °C; IR ν_max_ (cm^−1^): 3436, 1652, 1619, 1520, 1440, 1358, 1115, 1015, 976, 852, 818. ^1^H-NMR (CD_3_OD, 400 MHz): δ 4.47 (2H, s), 6.40 (1H, d, *J* = 15.6 Hz), 6.75 (1H, d, *J* = 8.0Hz), 6.90 (1H, dd, *J* = 8.0, 2.0 Hz), 6.98 (1H, m), 7.01 (1H, d, *J* = 2.0 Hz), 7.03 (1H, m), 7.12 (1H, m), 7.33 (1H, m), 7.43 (1H, d, *J* = 15.6 Hz). EI-MS *m*/*z* (%): 287 (M^+^, 100), 247 (35), 163 (95), 124 (90), 109 (50); UV(MeOH) λ_max_ (logε): 324 (4.37), 296 (4.30), 245 (4.28), 251(4.54) nm.

*N-(4-Methoxyphen)methyl caffeamide* (**MPMCA**): Solid; m.p.: 170–171 °C; IR ν_max_ (cm^−1^): 3283, 1653, 1613, 1520, 1447, 1374, 1116, 1009, 850. ^1^H-NMR (CD_3_COCD_3_, 500 MHz): 3.75 (3H, s), 4.44 (2H, d, *J* = 6.2 Hz), 6.49 (1H, d, *J* = 15.8 Hz), 6.81–6.94 (4H, m), 7.07 (1H, d, *J* = 1.6 Hz), 7.25 (2H, d, *J* = 8.8 Hz), 7.45 (1H, d, *J* = 15.8 Hz), 7.59 (1H, br. s, -NH), 8.17 (1H, s, -OH), 8.38 (1H, s, -OH). EI-MS *m/z* (%): 299 (M^+^, 7), 163 (100); UV (MeOH) λ_max_ (logε): 321(4.16), 295 (4.13), 284 (4.13), 245 (4.16) nm.

*N-Octyl caffeamide* (**OCA**): White solid; m.p.: 111–112 °C; IR ν_max_ (cm^−1^): 3286, 1642, 1588, 1520, 1363, 1277, 1112, 975, 811. ^1^H-NMR (CD_3_COCD_3_, 400 MHz): δ 0.84 (3H, t, *J* = 6.6 Hz), 1.24 (10H, m), 1.52 (2H, quin, *J* = 6.6 Hz), 3.30 (2H, q, *J* = 6.6 Hz), 6.47, 7.42 (each 1H, d, *J* = 15.6 Hz), 6.82 (1H, d, *J* = 8.2 Hz), 6.90 (1H, dd, *J* = 8.2, 1.8 Hz), 7.09 (1H, d, *J* = 1.8 Hz). EI-MS *m*/*z* (%): 291 (M^+^, 18), 220 (8), 193 (11), 178 (31), 163 (100), 145 (8), 135 (13), 128 (22), 117 (11), 98 (8), 89 (19), 84 (12); UV (MeOH) λ_max_ (logε): 322 (4.32), 294 (4.26), 238 (3.92), 219 (4.37) nm.

*N-Heptyl caffeamide* (**HCA**): White solid; m.p.: 126–127 °C; IR ν_max_ (cm^−1^): 3347, 1642, 1588, 1545, 1510, 1363, 1266, 1112, 975, 809. ^1^H-NMR (CD_3_COCD_3_, 400 MHz): δ 0.82 (3H, t, *J* = 6.6 Hz), 1.20 (8H, m), 1.52 (2H, quin, *J* = 6.6 Hz), 3.32 (2H, q, *J* = 6.6 Hz), 6.51, 7.48 (each 1H, d, *J* = 15.6 Hz), 6.83 (1H, d, *J* = 8.1 Hz), 6.92 (1H, dd, *J* = 8.1, 1.5 Hz), 7.11 (1H, d, *J* = 1.5 Hz). EI-MS *m*/*z* (%): 277 (M^+^, 42), 192 (10), 178 (32), 163 (100), 145 (12), 135 (20), 114 (14), 98 (8); UV (MeOH) λ_max_ (logε): 322 (4.23), 295 (4.17), 2.34 (4.16), 217 (4.30) nm.

### 4.3. Synthesis of Caffeate Derivatives

**Scheme 2:**
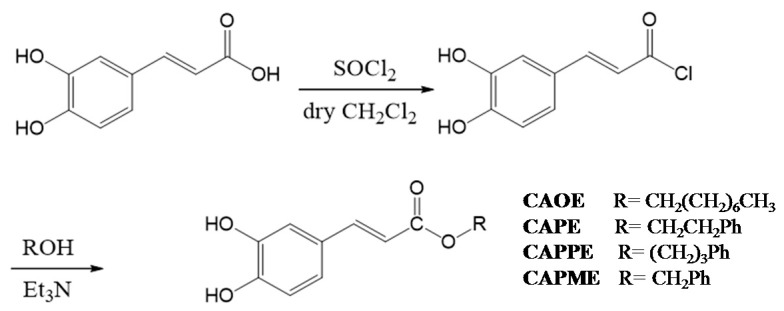
Synthesis of caffeic acid ester derivatives.

Caffeate derivatives were obtained as follows: Caffeic acid (200 mg) and thionyl chloride (4 mL) dissolved in dry dichloromethane (10 mL), were heated under reflux for 4 h. The solvent and excess SOCl_2_ was removed under vacuum, and then ROH (1.2 equiv.) in triethylamine (0.08 mL) was added dropwise under dry conditions. The reaction mixture was stirred for 24 h at ambient temperature, and then evaporated under vacuum. The residue was partitioned successively between EtOAc and H_2_O, and then the EtOAc layer was washed with 3N aqueous HCl then 10% NaHCO_3_ (aq.), dried over MgSO_4_ and concentrated under vacuum. The product was purified by column chromatography on silica gel. The final products (65%~75% yield) were recrystallized from acetone to obtain pure crystals. (Scheme 2).

*Octyl caffeate* (**CAOE**): White solid; m.p.: 98–100 °C; IR ν_max_ (cm^−1^): 3488, 3340, 1675, 1630, 1275, 1184, 972, 812; ^1^H-NMR (CD_3_COCD_3_): δ 0.85 (3H, t, *J* = 6.7 Hz), 1.26 (10H, m), 1.67 (2H, quin, *J* = 6.7 Hz), 4.16 (2H, t, *J* = 6.7 Hz), 6.23, 7.54 (each 1H, d, *J* = 15.9 Hz), 6.84 (1H, d, *J* = 8.2 Hz), 6.96 (1H, dd, *J* = 8.2, 2.0 Hz), 7.06 (1H, d, *J* = 2.0 Hz), 8.26 (2H, brs, -OH). EI-MS *m*/*z* (%): 292 (M^+^, 27), 180 (100), 163 (47), 145 (8), 136 (18), 134 (12), 89 (13).

*Phenethyl caffeate* (**CAPE**): Pale white needle-like crystals; m.p.: 126–128 °C, IR (KBr) ν_max_ 3400, 1709, 1605, 1519, 1352, 1282, 1192 cm^−1^; ^1^H-NMR (400 MHz, DMSO-*d*_6_) δ 2.95 (2H, t, *J* = 6.9 Hz, -OCH_2_CH_2_Ph), 4.32 (2H, t, *J* = 6.9 Hz, -OCH_2_CH_2_Ph), 6.23, 7.58 (each 1H, d, *J* = 15.9 Hz), 6.76 (1H, d, *J* = 8.1 Hz), 6.99 (1H, d, *J* = 8.2, 2.0 Hz), 7.03 (1H, d, *J* = 2.0 Hz), 7.22 (1H, m, H-4’), 7.18–7.30 (5H, m); EI-MS *m*/*z* (%): 284 (M^+^), 179, 161, 135 (100%), 117.

*Phenpropyl caffeate* (**CAPPE**): Yellow solid; m.p.: 116–118 °C; IR (KBr) ν_max_ (cm^−1^): 3482, 3327, 1671, 1629, 1597, 1179, 973, 809, 696; ^1^H-NMR (CDCl_3_): δ 2.01 (2H, quin, *J* = 6.8 Hz, -OCH_2_CH_2_CH_2_Ph), 2.72, 4.20 (each 2H, t, *J* = 6.8 Hz), 6.25, 7.55 (each 1H, d, *J* = 15.9 Hz), 6.85 (1H, d, *J* = 8.2 Hz), 6.98 (1H, dd, *J* = 8.2, 1.8 Hz), 7.08 (1H, d, *J* = 1.8 Hz), 7.10–7.30 (5H, m); EI-MS *m*/*z* (%): 298 (M^+^, 18), 180 (100), 163 (19), 135 (8), 118 (30), 117 (30), 91 (24).

*Phenmethyl caffeate* (**CAPME**): Light yellowish powder; m.p.: 150–152 °C, IR ν_max_ (cm^−1^): 3464, 3327, 1689, 1600, 1277, 1185. ^1^H-NMR (DMSO-*d*_6_, 400 MHz): δ_H_ 5.19 (1H, s), 6.32, 7.52 (each 1H, d, *J* = 15.9 Hz), 6.75 (1H, d, *J* = 8.1 Hz), 7.02 (1H, dd, *J* = 2.0, 8.1 Hz), 7.05 (1H, d, *J* = 2.0 Hz), 7.34–7.41 (5H, m), 9.20, 9.59 (each 1H, s, -OH). EI-MS *m*/*z* (%): 270 (9%, M^+^), 208 (19), 163 (50), 136 (33), 91 (100), 89 (32), 77 (19), 65 (20), 51 (18).

### 4.4. HPLC Determination of the CA Derivatives

**Figure 8:**
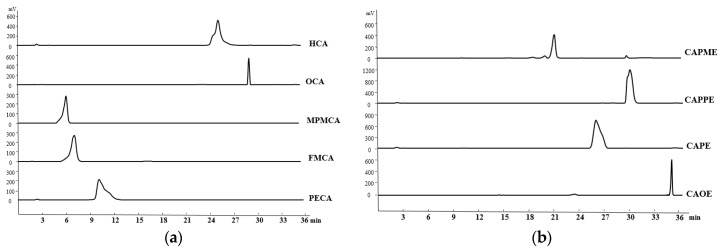
Retention time and purity analysis of caffeamide derivatives (**a**) and caffeate derivatives (**b**) by HPLC.

The purity of CA derivatives was measured by HPLC on a system (Ecom, Prague, Czech Republic) equipped with gradient pumps (Ecom LCP 4100), a UV detector (Ecom LCD 2084) and a LiChrospher^®^ 100 RP-18E (5 µm) HPLC column (125 mm × 4 mm i.d., Merck Millipore (Darmstadt, Germany). The mobile phase consisting of a mixture of solvent A (water/methanol, 98:2), and solvent B (methanol/acetic acid, 98:2) was run in the following gradient mode: 0–7 min, from 50% A to 40% A with a flow rate of 0.5 mL/min; 7–12 min, from 40% A to 30% A with a flow rate of 0.3 mL/min; and 12–28 min, from 30% A to 20% A with a flow rate of 0.2 mL/min. The UV detector was at 280 nm. Chromatographic processing was done using the Peak-ABC Chromatography Data Handling System. The purities of PECA, FMCA, MPMCA, OCA, HCA, CAOE, CAPE, CAPPE, and CAPME were 96.4%, 97.1%, 99.7%, 95.2%, 95.6%, 98.6%, 96.7%, 96.4%, and 98.3%, respectively (Figure 8).

### 4.5. Determination of the Viability of HepG2 Cells

The human HepG2 cell line (BCRC 60025) was obtained from the Bioresource Collection and Research Center (Hsinchu, Taiwan). Cells were maintained in DMEM (with 5.5 mM glucose) supplemented with 10% heat-inactivated fetal bovine serum (FBS, Gibco, Grand Island, NY, USA), penicillin (100 U/mL), and streptomycin (100 μg/mL) at 37 °C in a humidified atmosphere with 5% CO_2_. HepG2 cells (5 × 10^4^ cells/well) were cultured in 96-well culture plates and 24 h later treated with various concentrations of tested samples for the indicated times. Cell viability was determined using the 3-(4,5-dimethylthiazol-2-yl)-2,5-diphenyl tetrazolium bromide (MTT) assay. MTT (Sigma-Aldrich, St. Louis, MO, USA; 100 µL, 0.5 mg/mL) was added to each well and incubated at 37 °C for 3 h. The reaction was terminated by replacing the MTT-containing medium with 500 µL of dimethyl sulfoxide (Sigma-Aldrich), and the formazan salts were dissolved by gentle shaking for approximately 5 min at room temperature. The optical density (OD) of each well was measured at 595 nm using a microplate reader. Each assay was completed in triplicate wells, and each experiment was repeated three times.

### 4.6. Cytoprotective Effects of CA Derivatives

HepG2 cells (5 × 10^4^ cells/well) were seeded in 96-well plates. After 24 h, the culture medium was replaced containing various concentrations of caffeic acid and its derivatives (1, 5, 10, or 20 μM) or 20 μM silymarin (Sigma-Aldrich; as a positive control). Commercially available caffeic acid (Sigma-Aldrich) was used as a reference. After 24 h, the culture medium containing the samples was discarded, and the cells were treated for 20 h with 0.7 mM *t*-BHP (Sigma-Aldrich) to induce oxidative stress. Cell viability, as an indication of the cytoprotective effects of each of the samples, was then evaluated using the MTT assay.

### 4.7. Measurement of Lipid Peroxidation in HepG2 Cells

HepG2 cells were seeded in a 6-well plate at a density of 7.5 × 10^5^ cells/well. After 24 h, the culture medium was replaced with FBS-free medium containing different concentrations of CA and its derivatives (1, 5, 10, or 20 μM). After 24 h, the cells were treated for 24 h with 0.5 mM *t*-BHP to induce oxidative stress. After treatment, the cells were harvested and lysed by sonication. The lysates were centrifuged at 10,000× *g* for 10 min at 4 °C and used for the lipid peroxidation assay using TBARS assay. The absorbance was measured at 535 nm calibrated using solutions of authentic malondialdehyde (Sigma-Aldrich). The protein concentration was determined using the Bradford dye-binding method (Bio-Rad, Hercules, CA, USA). The results were expressed as nmol/mg protein.

### 4.8. Determination of Total GSH Level

HepG2 cells were seeded in 6-well plates at a density of 7.5 × 10^5^ cells/well. After 24 h, the culture medium was replaced with FBS-free medium containing various concentrations of CA and its derivatives (1, 5, 10, and 20 μM). After 24 h, the cells were treated for 24 h with 0.5 mM *t*-BHP to induce oxidative stress. After treatment, the cells were harvested and lysed using a sonicator. The lysates were centrifuged at 10,000× *g* for 10 min, and used for the determinations of protein and GSH. The GSH levels in HepG2 cells were determined by a commercially available GSH assay kit (Cayman Chemical, Ann Arbor, MI, USA). The results were expressed as nmol GSH/mg of protein.

### 4.9. Measurement of Mitochondrial Oxygen Consumption Rate

HepG2 cells were cultured at 1.5 × 10^4^ cells/well in XF24 cell culture plates (Seahorse Bioscience, Billerica, MA, USA). After 42 h, cells were treated with 20 μM of CA and its derivatives. After 6 h incubation, the media was replaced with 250 μL DMEM in the presence of 0.5 mM *t*-BHP for 2 h. Next, the media was exchanged with 675 μL XF Assay Medium-modified DMEM (Seahorse Bioscience) incubated at 37 °C without CO_2_ for 1 h. Mitochondrial oxygen consumption rate (OCR) was measured using an XF24 analyzer and software (Seahorse Bioscience). The cellular metabolic capacity and mitochondrial coupling were evaluated by injecting the following metabolic probes using the liquid injection ports on the XF instrument sensor plate: oligomycin, which inhibits the mitochondrial F_1_F_0_-ATPase, FCCP (carbonyl cyanide 4-trifluoromethoxy-phenylhydrazone), which is a protonophore that depolarizes the inner mitochondrial membrane, and the mitochondrial electron transport chain (ETC) inhibitors of complex I (rotenone) and complex III (antimycin A). The final concentrations of mitochondrial inhibitors were at 1 μM oligomycin, 1 µM FCCP, and 0.5 μM antimycin/rotenone. Basal respiration is the baseline oxygen consumption reading before compounds are injected. Maximal respiration represents the maximum OCR measurement value after FCCP injection. Spare respiratory capacity is calculated by noting the OCR response to FCCP.

### 4.10. Immunoblotting Analysis

HepG2 cells were grown in 100 mm culture dishes (2 × 10^6^ cells/dish) for 24 h, and then incubated with silymarin, CA, and CA derivatives (20 μM). After 24 h incubation, the cells were treated with 0.5 mM *t*-BHP to induce oxidative stress for 12 h. Cells were then harvested and washed with PBS. Whole cell lysates were prepared in a lysis buffer (Cell Signaling, Beverly, MA, USA) containing 10 mM phenylmethylsulfonyl fluoride (PMSF). The cell lysates were sonicated and cleared by centrifugation at 4 °C, 14,000× *g* for 10 min. Whole cell lysates were examined for HIF-1α expression. The nuclear proteins from harvested cells were prepared using the nuclear fractionation kit (Imgenex; San Diego, CA, USA) and were examined for Nrf2 expression. Protein concentrations were determined by DC protein assay (Bio Rad). Aliquots of the cell lysates (each containing 20 μg of protein) or nuclear protein (10 μg) were boiled for 5 min and electrophoresed on a 10% sodium dodecyl sulfate (SDS)-polyacrylamide gel. Following SDS-polyacrylamide gel electrophoresis, proteins were transferred to PVDF membranes. Membranes were blocked by incubation in gelatin-NET buffer at room temperature, and then incubated with 1:1000 dilution of primary antibodies of Nrf2 (GeneTex, Irvine, CA, USA), HIF-1α (GeneTex), lamin B (GeneTex) and β-actin (Sigma-Aldrich), followed by horseradish peroxidase-conjugated secondary antibody according to the manufacturer’s instructions. The immunoreactive proteins were detected using the enhanced ECL chemiluminescence Western Blotting Detection System (ChemiDoc XRS, Bio-Rad). Signal strengths were quantified using densitometric program (Image Lab, Bio-Rad).

### 4.11. Statistical Analysis

All data are presented as means ± SD. Statistical analyses were performed using the SPSS 19.0 statistical package (Chicago, IL, USA). The data were evaluated for statistical significance with the one-way ANOVA and Duncan multiple comparison test. A *p* value of <0.05 was considered statistically significant. The concentration of tested samples required to inhibit 50% of the activity under the assay conditions was determined from dose-response curves and defined as the IC_50_ value.
